# New developments in fabrication of high-energy-resolution analyzers for inelastic X-ray spectroscopy

**DOI:** 10.1107/S0909049511001828

**Published:** 2011-03-10

**Authors:** Ayman H. Said, Harald Sinn, Ralu Divan

**Affiliations:** a Advanced Photon Source, Argonne National Laboratory, Argonne, IL, USA; b European XFEL, Notkestrasse 85, 22607 Hamburg, Germany; c Center for Nanoscale Materials, Argonne National Laboratory, Argonne, IL, USA

**Keywords:** high-energy-resolution analyzers, inelastic X-ray scattering spectroscopy

## Abstract

In this work new improvements related to the fabrication of spherical bent analyzers for 1 meV energy-resolution inelastic X-ray scattering spectroscopy are presented. The new method includes the use of a two-dimensional bender to achieve the required radius of curvature for X-ray analyzers. The advantage of this method is the ability to monitor the focus during bending, which leads to higher-efficiency analyzers.

## Abstract 2

New improvements related to the fabrication of spherical bent analyzers for 1 meV energy-resolution inelastic X-ray scattering spectroscopy are presented.

## 1. Introduction

Typical inelastic X-ray spectrometers around the world have two critical components for achieving an energy resolution of the order of meV: the high-resolution monochromator and the analyzer. The monochromatization of X-rays to 1 meV energy bandwidth can be achieved using different schemes by using either a single high-order silicon Bragg reflection in extreme backscattering geometry (Verbeni *et al.*, 1996 ▶; Baron *et al.*, 2001 ▶) or a multireflection in-line monochromator (Toellner, 2000 ▶; Toellner *et al.*, 2006 ▶; Ishikawa *et al.*, 1992 ▶; Mooney *et al.*, 1994 ▶).

Unlike monochromators, which have to monochromatize a beam with a very narrow angular divergence of a few microradians, analyzers have to accept, focus and maintain the energy resolution of the scattered radiation with angular divergence of the order of 10 mrad. Therefore, the fabrication of analyzers for meV spectroscopy has been a challenge. Several developments have taken place (Graeff & Materlik, 1982 ▶; Dorner & Peisl, 1983 ▶; Burkel *et al.*, 1987 ▶; Sinn *et al.*, 2002 ▶; Verbeni *et al.*, 2005 ▶; Collart *et al.*, 2005 ▶; Masciovecchio *et al.*, 1996 ▶).

In this work we present an improved method of fabricating analyzers for inelastic X-ray spectroscopy with 1 meV energy resolution. The method is based on using a two-dimensional bender and an optical alignment method to obtain an optimized focus. The use of a bender has been demonstrated previously (Sinn *et al.*, 2002 ▶). The advantage of the new method is the ability of monitoring the focus during bending in addition to maintaining the optimized focus. The described method has a higher success rate of producing analyzers with similar efficiency and similar performance. The development, implementation and testing were carried out at the HERIX spectrometer at sector 30 at the Advanced Photon Source.

## 2. Fabrication

The fabricating steps of spherical analyzers for high-energy-resolution spectroscopy are summarized in the following steps.

(i) *Preparation of silicon wafer.* The silicon wafers are sliced from a high-electric-resistance ingot (>50 kΩ). The normal to the wafer surface is parallel to the Si(111) direction. After that the wafers are rounded, etched in an HNO_3_ and HF acid mixture and double-side polished to optical grade (roughness is better than 10 Å RMS and flatness is better than six fringes).

(ii) *First dicing step.* For Si wafers ranging in thickness between 4 and 6 mm it is too difficult to dice all the way through the crystal using a 50 µm-wide blade. The dicing is performed in two steps. First, one side of the wafer is cross-diced 3–4 mm deep with a 200–300 µm-wide blade. Second, the other side of the wafer is cross-diced after bonding to a glass wafer using a 50 µm-wide blade. For thinner Si wafers, less than 4 mm, it is possible to dice the wafer all the way through using a 50 µm-wide blade.

(iii) *Anodic bonding.* For the prediced wafer the diced side is bonded to the glass. Anodic bonding is a well known technique in the semiconductor industry, and works between Si and a glass containing a high concentration of alkali metal oxides, *e.g.* Pyrex (brand corning 7740). The thickness of the glass is 2 mm and the diameter is 125 mm. More details about the minimum thickness of the glass disc that can be used are described elsewhere (Sinn *et al.*, 2002 ▶). Both surfaces of the glass wafer are polished to a surface roughness of better than 0.1 µm. Pyrex 7740 has a similar thermal expansion as silicon at room temperature and also at higher temperature, which is critical for bonding. Bonding requires a high voltage (300–1500 V) and high temperature (470–725 K). Bonding is performed inside a clean room to avoid having dust particles between the wafers, which causes breaking of the wafers upon bonding. By applying the voltage across the wafers at higher temperature, the Na^+^ ions in the glass move away from the Pyrex/Si interface leaving negatively charged oxygen ions at the interface. Subsequently, the electrostatic force pulls the Si and glass together to form the bonding. The high voltage at constant current is applied for about 50–60 min. The current is kept at 3 mA during bonding. For the undiced Si wafer we used a pre-diced glass wafer. The dicing of the glass disc is performed using a 100 µm-thick Resinoid blade. The depth of the cuts are 200 µm. The distance between the grooves depends on the analyzer. The distance matches the required pixel size.

(iv) *Second dicing step.* The silicon wafer is cross-diced by matching the grooves on the glass disc for the thin wafer or matching the groves on the Si wafer. Dicing is carried out using a 50 µm blade through the entire thickness or remaining thickness of the Si wafer without leaving any back wall, which results in free-standing Si pixels. It takes two to three days to complete this dicing step. The groove width after dicing is about 75 µm. The final width of the groove varies as a function of cutting speed and rotation speed.

**Figure 1:**
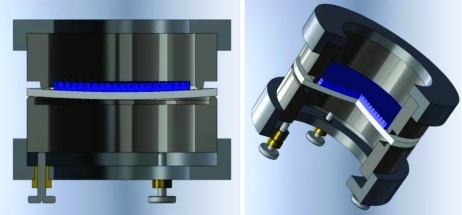
Schematic for the two-dimensional bender.

**Figure 2:**
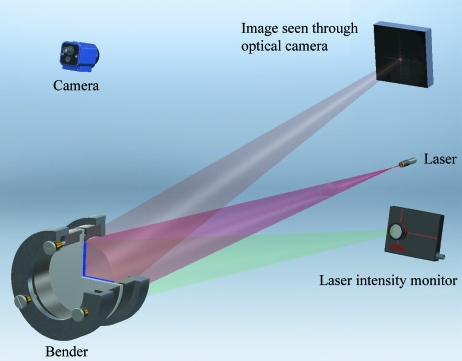
Schematic of the optical set-up used in the focus optimization.

**Figure 3:**
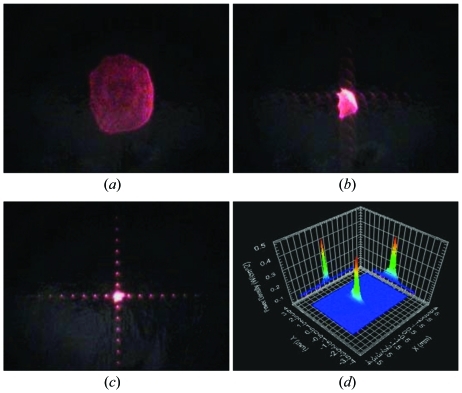
(*a*) The image formed on a white board from the analyzer by slightly tightening the screws on the bender, (*b*) a change in focus as screws are tightened further, (*c*) an optimized focus, (*d*) the intensity profile of the focused beam.

(v) *Primary etching.* The diced/bonded wafer is etched using a 30% potassium hydroxide (KOH) solution at 353 K to remove the stress developed during the dicing process. The etching process takes 12 min. About 25 µm is removed from each side of the pixels. Because KOH solution is highly anisotropic (Seidel *et al.*, 1990 ▶), the solution tends to have a very minimum effect on the Si (111) planes, which protect the reflecting polished surface of the diced silicon wafer.

(vi) *Bending and focus optimization.* (*a*) The Si/glass wafer is placed inside the two-dimensional bender, which has been described previously in more detail elsewhere (Sinn *et al.*, 2002 ▶; Fujii *et al.*, 1982 ▶). The two-dimensional bender consists of two metal cylinders with a doughnut-shaped contact surface having different diameters for the upper and lower cylinders (Fig. 1 ▶). The two stainless steel rings were manufactured with a high-precision diamond-tool flycutter machine and were kept centered and parallel to each other by an outside guidance ring. The upper ring is fixed in motion; the motion of the lower ring can be controlled by three or six mechanical screws. We find three screws 120° apart to be sufficient to obtain a good curved surface. To allow a smooth sliding of the glass during the bending process a thin layer of Kapton foil and/or rubber were put between the metal rings and both sides of the glass wafer.

(*b*) The Si wafer inside the bender is placed at a distance *R* from a laser source; *R* is equivalent to the distance between the sample and the analyzer. In our case *R* = 9090 mm.

(*c*) The laser beam is aligned to be perpendicular to the surface of the Si wafer and to be in the center.

(*d*) An adjustable short-focal-length lens is used in front of the laser source to obtain a divergent beam, which covers the whole analyzer at distance *R*.

(*e*) The distance *R* is adjusted to be between the focal length of the lens and the analyzer.

(*f*) A white board is mounted a few millimeters above the laser beam. The white board is placed at a distance *r* from the focal point of the laser, as shown in Fig. 2 ▶; *r* in our case was 180 mm.

(*g*) A laser beam profiler is mounted a few millimeters below the laser beam in the same vertical plane with the white board, as shown in Fig. 2 ▶.

(*h*) The mechanical screws are tightened on the bender slightly, carefully and equally to start focusing the reflected beam from the Si wafer onto the white board. The shape of the image formed by the analyzer changes as the screws are tightened. The change in the focus as a function of bending is shown in Fig. 3 ▶.

(*i*) The angles of the bender are adjusted to direct the reflected beam on the white board, as shown in Fig. 2 ▶.

(*j*) The mechanical screws are tightened some more to obtain a good focus, which is indicated by the interference pattern caused by the two-dimensional pixels array, as shown in Fig. 3(*c*) ▶. The bender tends to relax over time, which causes deterioration of the focus. A constant optimization of the focus is needed. The relaxation process slows down as a function of time. A minimum of two weeks is needed to ensure stability of the bending.

(*k*) The angle of the bender, in the vertical plane, is changed to point the focus on the beam intensity profiler, as shown in Fig. 2 ▶. Fine-tuning can be achieved by maximizing the intensity and optimizing the profile of the focus. The profile of the focus is shown in Fig. 3(*d*) ▶.

(*l*) To fix the bending radius after reaching satisfactory results with the bending, a concave lens is glued to the bent wafer *via* the following steps. The bender is set up on a table with the glass wafer facing up (Fig. 4*a* ▶). A concave Pyrex (7740) glass lens is glued to the bent glass wafer inside the bender using a low-viscosity UV glue (EPO-TEK OG142-87). The thickness of the glass lens is 15 mm, the radius of curvature is 8909 ± 5 mm and the surface roughness is better than 10 Å RMS with surface quality 60/40 scratch/dig. The lens is diced with a 300 µm blade to prevent air bubbles between the surfaces. The glue has to be distributed equally over the surface of the concave lens, and the thickness of the glue layer is about 20–25 µm. A thicker glue layer can lead to changes of the shape of the bent wafer upon curing owing to weight loss of the glue. After that, the bender is placed under a UV lamp (400 W) for 4–5 min to allow the glue to cure (Fig. 4*b* ▶). Using a UV glue is critical to prevent any relaxation of the bender after obtaining a good focus.

(vii) *Final etching.* The analyzer is removed from the bender and etched using a solution containing 93% HNO_3_ acid and 7% HF acid. The total etching time is about 2–5 min; the etching was interrupted periodically by rinsing the analyzer in water after 1 min of etching to slow down the etching process to avoid breaking the analyzer. During etching, the glue layer between the glass lens and the glass wafer should be protected from the acid solution. A Teflon holder was used for this. The final analyzer is shown in Fig. 5 ▶.

## 3. Analyzer tests and resolution function

We prepared two analyzers using the method described above. The thicknesses of the silicon wafers were 3.3 mm. The initial sizes of the pixels after dicing were 1.17 mm × 1.17 mm and 1.07 mm × 1.07 mm. The final sizes of the pixels after etching were 1.09 mm × 1.06 mm and 0.95 mm × 0.94 mm. Upon removing the analyzer from the bender we mounted it at distance *R* from the laser and compared the focus with that before gluing. This ensures the quality of the analyzer as a final measure before testing with X-rays.

**Figure 6:**
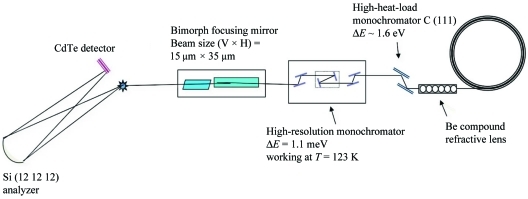
Schematic of the HERIX spectrometer. The distance between the sample and the analyzer is 9090 mm and the vertical distance between the center of the detector and the direct beam is 3.5 mm. The high-heat-load monochromator consists of two water-cooled diamond C (111) crystals. The high-resolution monochromator is a six-reflection crystal in-line monochromator with 1.1 meV energy bandwidth. The energy scans were carried out by rotating the crystal. The distance between the sample and the detector is 180 mm.

**Figure 7:**
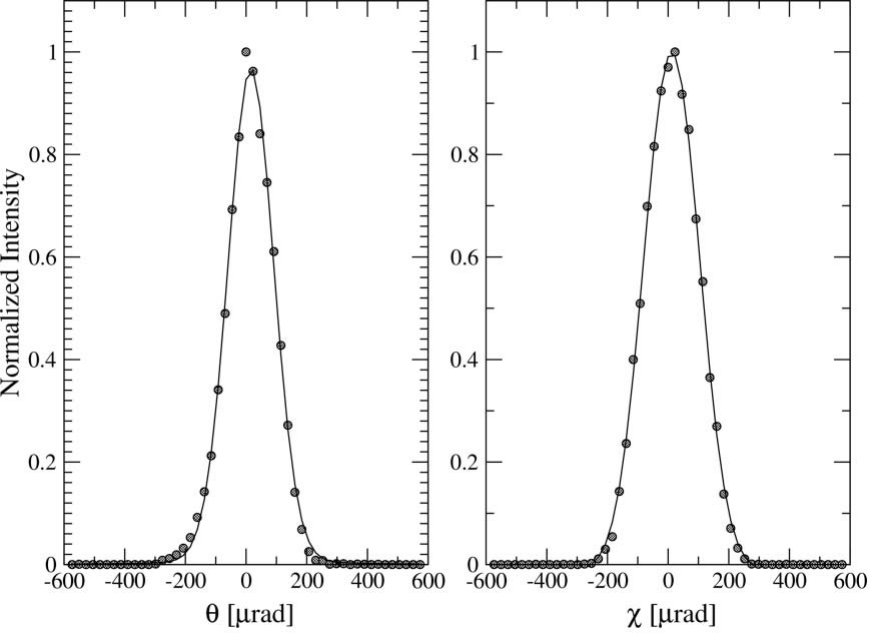
Angular scans for the analyzer measured by the CdTe detector.

The actual tests of the analyzers were carried out using the HERIX spectrometer at beamline 30 ID-C. A schematic view of the HERIX spectrometer is shown in Fig. 6 ▶. The resolution function is measured by looking at the elastic scattering from a Plexiglas sample (10 mm-thick) at room temperature. The elastic scattering is collected at *Q* = 10 nm^−1^, which corresponds to the maximum intensity of the structure factor. The incident energy was 23.724 keV, which corresponds to the backscattering energy of the Si (12 12 12) reflection. After aligning the analyzer to focus the scattered radiation from the sample to the detector, the focus is measured by scanning the rotation angles of the analyzer θ (vertical plan) and χ (horizontal plan), as shown in Fig. 7 ▶. The FWHM of the θ angular scans for both analyzers were 233 µrad and 179 µrad, and the FWHM of the χ angular scans for both analyzers were 235 µrad and 210 µrad. The measured angular width is a measure of the analyzer slope error convoluted with the effect of pixel size.

**Figure 8:**
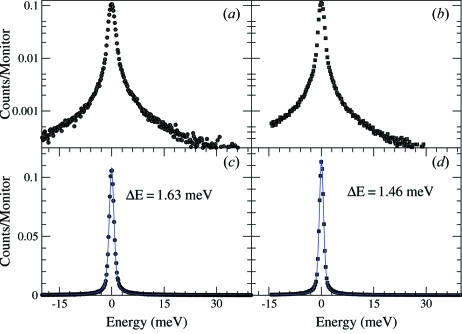
Resolution function measured from two analyzers. (*a*) and (*b*) Logarithmic plots of the measured resolution functions. (*c*) and (*d*) Raw resolution and the pseudo-Voigt fits; the black symbols are the data and the blue lines are the fit. The estimated error bar in the measured resolution is about 0.1 meV.

**Figure 9:**
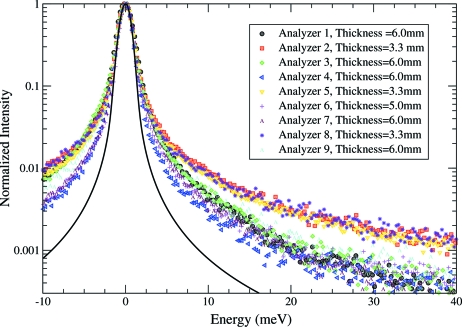
The resolution function for nine analyzers with different thicknesses; analyzers 2, 5, 6 and 8 were prepared by the method described in this paper, the other analyzers were prepared differently. Clearly the tails of the elastic peaks are lower for the thicker analyzers than for the thinner ones. The solid line is the theoretical curve.

The energy scans were carried out by rotating the cryogenically cooled channel-cut crystal of the high-resolution monochromator. The bandwidth of the incident energy was 1.1 meV. The resolution functions for both analyzers are shown in Fig. 8 ▶. A pseudo-Voigt function was used to fit the resolution function to determine the FWHM. The fitted curves are shown with solid lines in Figs. 8(*c*) and 8(*d*) ▶. The resolution function for analyzer 1 is shown in Figs. 8(*a*) and 8(*c*) ▶ on logarithmic and linear scales, respectively. The resolution function for the second analyzer is shown in Figs. 8(*b*) and 8(*d*) ▶. The measured intensity was normalized to the monitor intensity before the sample to be able to compare the relative efficiency of the analyzers. The measured relative efficiencies from both analyzers prepared by the method described were comparable with the highest relative efficiencies measured from analyzers prepared previously by different methods. The overall energy resolution (FWHM) was different between the two analyzers. The different widths might be caused for different reasons such as the etching time inside the HNO_3_–HF solution, which differed by 1 min. Analyzer 1 was etched for 2 min and analyzer 2 was etched for 3 min. Also, the possible temperature gradient on the analyzer can contribute to the difference in the energy resolution. The tails of the elastic peak were higher than the calculated ones, as shown in Fig. 9 ▶, which is partially due to the thickness of the silicon. A third thicker analyzer (5 mm-thick) was prepared by the same method for comparison. As shown in Fig. 9 ▶, all thick analyzers, regardless of the way they were made, have better peak tails than the thinner ones and are closer to the calculated curve, shown in Fig. 9 ▶ as a solid black line.

## 4. Conclusion

The procedure for making analyzers for high-energy-resolution inelastic X-ray scattering spectroscopy described in this work will enable the production of these analyzers with very similar efficiencies. The ability to monitor the focus during bending is the key to achieving this goal. By dicing the silicon in a two-step procedure, thicker silicon substrates can be used, which leads to reduced intensity in the tails of the resolution function.

## Figures and Tables

**Figure 4:**
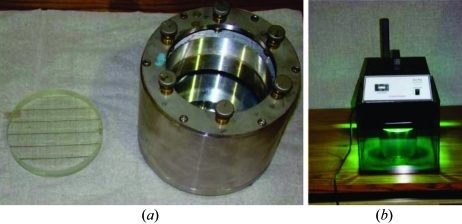
(*a*) The bender and the concave lens. (*b*) The bender exposed to the UV light source.

**Figure 5:**
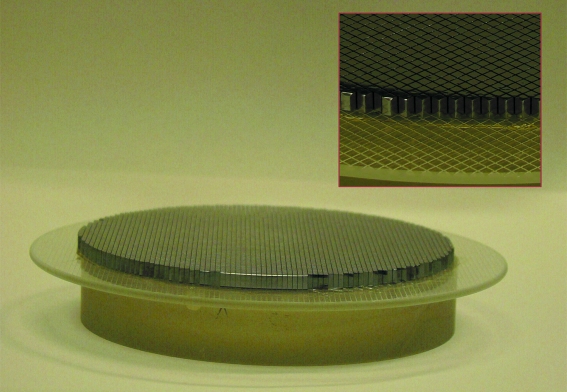
The complete analyzer. The inset is an enlargement of a small part of the analyzer to show the individual pixels.
